# Hospital and Institutional News

**Published:** 1920-05-22

**Authors:** 


### HOSPITAL AND INSTITUTIONAL NEWS.

### PRINCE ALBERT AS HOSPITAL PRESIDENT.

Prince Albert, who has accepted the position of President of the Queen's Hospital for Children, Hackney Road, entered upon his official duties at the annual meeting of the Governors, held on the 14th inst. at Shoreditch Town Hall. With charming diffidence, the Prince said that he did not care to undertake criticism of his hospital, but he felt that the out-patient department was strikingly inadequate, and he was glad to learn that when funds and working conditions improved a scheme of enlargement would be undertaken. But the Queen's, like so many other hospitals, is passing through a stage, strictly limited in extent we hope and believe, when cash for current needs stands in seeming importance much in front of funds for extensions. The expenditure of the Queen's rose in one year (1918-1919) from £21,000 to £27,000, and unless help comes speedily sixty-two beds are to be shut down. This must not be, and Prince Albert's advocacy will go a long way to prevent the catastrophe.

### THE SATURDAY FUND PROGRAMME.

If Mr. Philip A. Inman, the Secretary of the Metropolitan Hospital Saturday Fund, is the subject of a friendly catechism by a special correspondent on another page of this issue, he is to be congratulated upon the questions which, in his turn, he addresses to the public in a batch of current leaflets. For the Saturday Fund reprints an article by Sir Philip Gibbs which appeared in the *Daily Chronicle* on January 12 last, and has issued a manifesto which is encouraging. Sir Philip Gibbs declares, it is true after a momentary hesitation, that "the old system of voluntary contributions must go on," and that at once, and calls attention to the "definite programme" which the Council of the Fund has drafted to meet the urgent needs of the case. The manifesto, alluding to this programme, states that "the time is opportune for a great forward movement in the hospital world." The benefits open to subscribers are thus shortly printed under seven heads. These are followed by an application form for collecting-cards, sheets, and boxes, together with a contribution form. The latter asks for a special gift and for an annual subscription. The circulation of this manifesto presumably is the chief item of the programme, which is good so far as it goes. The special correspondent already referred to examines the present position of the fund by the light of its published statistics. The questions which this examination leads him to ask suggest a methodical plan for making the present organisation more thorough. There is no reason why every practical scheme should not be tried, and we trust that none will be neglected.

### HOSPITAL TO PAY INCOME TAX.

The House of Lords unanimously allowed the appeal of the Surveyor of Taxes against decisions of the King's Bench Division and Court of Appeal in Ireland, as to the manner of assessing the Governors of the Rotunda Hospital, Dublin, to income tax in respect of the Rotunda Rooms which the hospital lets for concerts, cinema entertainments and the like. It was contended by the Governors that, as by the Statute regulating the hospital, all profits are to be applied for the maintenance of the charity, the hospital, with the receipts from the rooms, must be regarded as one undertaking which yielded no profit, and accordingly was not liable to taxation. But the Lords held that the argument was not maintainable. No doubt the hospital, like other charities, yielded no profit, but if the Governors, in the course of their management, carried on a profitable business, the profits of that business would be subject to taxation. The case has reached the highest tribunal of the land, and there is nothing left but to pay the tax, although the position is a curious one. If a hospital let part of its buildings, or an adjoining building belonging to it, for continuous use as a residence or a business house, it could recover the tax deducted for the rent. But if a hospital lets such a building for an afternoon or an evening for the purposes of an entertainment, tax is payable upon the fees obtained if during the year the aggregate reaches a taxable amount.

### IN HONOUR OF THE EARL OF ATHLONE.

Our Queen Mary's brother, the Earl of Athlone, is well known to our hospitals, especially at the Middlesex, where he is Chairman of the weekly board. He is also a Vice-President of the National Hospital, Queen Square. Last week a number of the most prominent surgeons of the day dined together at Claridge's Hotel, and the Earl of Athlone was the guest of honour. A feature of the dinner was the manner in which the guests were grouped, each table bearing a distinctive flower, instead of a table number, and by means of this floral indicator the guests were able to find their places. Sir John Bland Sutton presided over the gathering and, as was to be expected of one of our wittiest speakers, amused the guests greatly.

### MEMORIAL TO SIR JOHN WOLFE BARRY.

A General Council is being called together by the Institution of Civil Engineers to deal with the preliminaries in connection with a memorial to the late Sir John Wolfe Barry in order to carry into effect the proposal to place a window in Westminster Abbey. Permission has been obtained for the erection of the window, and the Abbey authorities have instructed an artist to prepare a design for consideration. The memorial is a very appropriate one, for the great civil engineer was associated with Westminster in so many ways, amongst others as Chairman of Westminster Hospital.

### ACTION OF THE BRITISH HOSPITALS' ASSOCIATION.

The representative Committee for London of the British Hospitals' Association has gone quickly to work by means of a form of questions which has been sent to the Metropolitan hospitals in order to ascertain the financial position as at the end of April. The inquiry is simple and direct, and no difficulty should be found in answering them rapidly and accurately. A similar committee has been formed in Birmingham for the Midlands, and has held its first meeting at Wolverhampton. The London Committee is a strong one, and is under the chairmanship of Viscount Hambleden; it includes the following: Viscount Knutsford, the Hon. Sir Arthur Stanley, representatives of St. Bartholomew's, Guy's, and Middlesex Hospitals; Lord Winterton, Mr. John Murray, and Mr. C. E. L. Lyle. It is hoped to form strong area committees of the British Hospitals' Association throughout the country, the areas coinciding with the regional areas of the Ministry of Pensions.

### "PRO ARIS ET FOCIS."

Under the above title the *London Hospital Gazette* for May issues a supplement containing the names of all connected with the London Hospital who served in the Great War, 1914-1919. Comprising past and present students, including members of hospital and college staffs and voluntary workers and civilians, members of nursing staff, and members of house-committee and lay staffs, the list extends over 33 closely printed pages and must contain more than 1,500 names. On three sad pages we find the records of some 112 Londoners who have not returned from the war. The list concludes with particulars of the honours bestowed during and after the war upon the doctors, students, nurses, and general staff of the London Hospital. We notice that about 130 nurses were awarded the Royal Red Cross, and about the same number of Military Crosses were conferred on the doctors, etc. There are, of course, a number of other British distinctions mentioned, and also various decorations from the Allies, including Belgium, China, France, Greece, Italy, Portugal, Russia, Serbia, Siam, and the United States of America.

### NATIONALIZATION.

The latest recruit of importance to periodical journalism is *Blackfriars*, a shilling monthly review. The May number contains a striking article by Mr. Hilaire Belloc upon the subject of "Nationalization." Although the word "hospital' is not mentioned, the article deserves attention by institutional workers, because the object of the essay is to explain the origin, philosophic basis, intention, and probable effect of the present movement to nationalize industry and institutions. The ethics of the nationalization of industry do not concern us here; but, since the article is concerned with principles rather than with detail, those who support the voluntary system of hospital finance may study from the essay in question the nature of the principle which they are opposing. We do not propose to summarise the argument nor to condense the exposition, but merely to invite attention to both. A study of the argument will do something also to justify Mr. Belloc's contention that the essay would not have been accepted by the lay commercial Press, which, as Sir Phillip Gibbs points out in another article of the same number of *Blackfriars*, is largely at the beck of financial interests, and is apt to manipulate the presentation of news at their behest. We note this point, not because it is a new one, but because, perhaps, the chief value of technical journalism is to steady general opinion in regard to its own subject, and to correct erroneous impressions by the regular supply of accurate facts. For these reasons we commend the article in question to the attention of all who are convinced that the voluntary principle must be maintained in English life.

### WHAT BECOMES OF GOVERNMENT FORMS?

During the war there was a strong suspicion that the greater part of the forms which were filled up by hospital authorities for the War Office, the Coal Controller, the Food Officer, and other of our benevolent tyrants, were filed for future reference and that that was the end of their usefulness. Anyhow, it often happened that when certain periodical forms were not sent in, or were discontinued altogether, they apparently were never missed. The Ministry of Pensions is becoming at the present time addicted strongly to the form habit, and if they get all they ask for as often as they ask for it there must be hundreds of tons of forms in the possession of the Ministry. An amusing example of this form of Government acquisitiveness comes from Cromer. In October 1914 a "First Line" Hospital was set up in charge of Lady Layland-Barratt. The hospital was closed on December 10, 1917. While working the hospital was required to furnish quarterly "employment returns" to the Board of Trade, and did so. When it closed, two and a half years ago, the Board was notified. But demands for "employment returns," with forms in duplicate, continue to arrive at the house which was once a hospital. The latest asks for returns up to April 24, 1920, and asks for an answer by April 26.

### WHAT HAPPENS TO THE MEDICAL STUDENT?

Mr. Corner has carried out an inquiry into the careers of ten years of students entering St. Thomas's Hospital. He finds that every year about eleven per cent. of the annual entry "drop out from work in the profession," and about two die. In every year eighty-seven per cent. of students persevere and get qualified. Sir James Paget once made a wider classification and published his findings in a paper written in 1869 entitled "What becomes of Medical Students?" the figures being based upon the records of men at St. Bartholomew's. The reading is not cheering to the medical aspirant, but possibly things have improved in fifty years. Distinguished success was achieved by 2.3 per cent., considerable success by 6.6, fair success by 50.7, very limited success by 12.4, and those who failed were 28 in every hundred. Failure was due either to death (after qualification 8.7 per cent., and before qualification 4.1 per cent.), or to giving up the profession (15.2 per cent.). If success can be construed as making a living the National Health Insurance Act must have improved the incidence since Sir James Paget's day.

### LET US UP AND BE DOING.

While some hospital treasurers and secretaries content themselves by sitting down and groaning about hard times, others are always on the look-out for fresh means of making the work of the institution known by getting at the individual who is waiting to be interested and will not come up to the scratch until he realises that he himself, and not the crowd, is being addressed. Faint hearts must work out their own salvation, but, for the alert and enthusiastic, the news of what is being done elsewhere is always helpful. Our own Lady Mayoress has addressed a letter to "Dear Kind Ones" in connection with the dire necessity of the Metropolitan Hospital. Such an appeal, departing from the stereotyped form, is sure to obtain success. The Alexandra Hospital for Tuberculous Children has secured a champion in the person of *John Bull*, who will at least obtain the attention of the multitude. At Saltash the S. Barnabas Hospital and Nursing Home have organised a jumble sale, an amusing expedient which is likely to succeed where the old familiar dry-as-dust appeal will fail. The Royal Gwent Hospital makes a timely appeal to women, and tells them that helping hospitals is an opportunity of establishing their citizenship. At Cardiff the Lord Mayor's Hospital Fund makes use of the favourite game of "rugger" to secure some thousands of entirely new supporters, while at Exeter there is an appeal for eggs, potatoes, and other vegetables, which has elicited gifts in value between £250 and £300. At Bristol the Children's Hospital has benefited to the extent of £1,070 by a house-to-house collection during which 43,000 calls were made. This is the way to do it—get at the individual and all the money required will be forthcoming. It needs a little ingenuity and more than a little hard work, but think how the circle of supporters is gradually and steadily increased!

### THE VENTILATION OF CINEMA HOUSES.

Recently the Deptford Borough Council made representations to the Ministry of Health as to the necessity for improved ventilation at cinemas and theatres. Now the Ministry replies that it has come to the conclusion that no permanent or satisfactory improvement in the ventilation of such places will be effected by emergency regulations in times of epidemics, adding that it is a matter requiring time and steady pressure by local authorities in conjunction with the local licensing authority. Nevertheless, the Ministry adds that it is having inquiries made as to what can be regarded as a reasonable minimum of ventilation for these places.

### A PENNY IS STILL A PENNY.

Mr. Knowles, the secretary of the Manchester and Salford Hospital Saturday Fund, uses figures in a most striking way to support his appeal for Hospital Saturday. He tells us that there were 59,834 accident cases treated last year by the hospitals, the cases being almost equally divided between the Royal Infirmary, Salford Royal Hospital, and Ancoats Hospital. In the twenty-one Manchester institutions there were 32,637 in-patients and 304,654 out-patients during the twelve months. But Mr. Knowles points out that while work and expense grow at the hospitals, the workman's penny is still a penny, although his wages have been materially increased. Whereas Manchester gave only £3,665 in 1919 the Leeds people contributed £25,000. Mr. Knowles hopes that the old-fashioned belief that a penny is the classic coin for a hospital box will die quickly and that the workpeople of Manchester this year will realise that their responsibilities, as well as their privileges, are considerably changed.

### THE GUEST HOSPITAL, DUDLEY.

The good-will of industry, and the term includes both employer and employed, is especially at this time necessary to a hospital in an industrial centre, such as the Guest Hospital at Dudley, and from all accounts the hospital and many potential supporters have not been, as our American friends would say, "brought together." Some correspondence has been published between Mr. Arthur Bird, the secretary, and Sir George Bean, who, in the secretary's words, is looked upon as "the leader of the commercial and general life of the town." Mr. Bird alludes to the different financial position and asks Sir George to give the employers a lead. But Sir George Bean frankly says that he and many others are not satisfied with the administration of the hospital. It is thought that with regard to the committee and the honorary surgical staff, appointments are made in a hole-and-corner manner, and the best men are not always chosen. Any investigation of complaints, it is further said, is carried on under conditions that are one-sided and wholly in the interests of the staff. The *x*-ray and electrical apparatus is inefficient, and there is nobody at the hospital qualified to operate it. These things must be cleared up, and we suggest that a small committee of inquiry be set up with members chosen in equal number by the hospital committee and Sir George Bean. Every hospital needs all the friends it can get and the democratic spirit of the age must be recognised at Dudley as elsewhere.

### HOSPITAL'S HEAVY LIABILITY.

The governors of the Bolingbroke Hospital, Wandsworth Common, have had a trying year, and, although their liabilities have not increased, they are still faced with a debt of £8,426 17s. 11d. Unfortunately, the hospital has not received the financial support of the working classes, who are the chief patients, but a scheme is to be prepared whereby the employers of the district are to be invited to co-operate with the governors in obtaining from their workpeople more adequate support. Founded in 1880 by the late Canon Erskine Clarke as a home for sickness, the hospital, incorporated in 1896, has gradually grown until it now contains sixty beds, which, it is hoped, by the time the jubilee is reached will have increased to 100 beds. The hospital has a special interest to those interested in radium research work, for in 1885 Mr. Cecil Lyster became associated with it, and for fifteen years whilst he remained there he commenced the radiological research work which cost him his life. The Mayors of Wandsworth and Battersea have done much to assist the hospital, and have still open funds for subscriptions; their special appeal during the year resulted in £1,914 being raised.

### A CERTAIN LIVELINESS IN EAST LONDON.

Plenty of energy appears to be expending to make the appeal of the Metropolitan Hospital a success. There is a map designed to show where the bombs were dropped in London, reproduced by the Special Appeal Committee, of which Mr. Sydney N. Young is chairman, from the *Daily Mail*. This shows the residents in the crowded area served by the hospital not only that it was so situated as to be the bull's-eye of the target offered by London to the German raiders, but also how much work the Metropolitan Hospital had to do in relieving the victims of the bombers. One hundred thousand or more of these leaflets have been distributed in the district, and one could only wish that the site of the hospital could have been indicated in red ink upon the map. The Mayoresses of Bethnal Green, Hackney, Islington, Shoreditch, and Stoke Newington sign the appeal. We hope this also means that the work is being decentralised, that each mayoralty has its own committee, its own organisation, so that in the end there shall be no house nor street in these districts which has not been individually approached, perhaps even personally visited. Decentralisation under a chief executive, which would presumably be the Special Appeal Committee, can do this; but it cannot be done thoroughly in any other way. All the local newspapers should be enlisted, and each of them should present the case in a different way. No cinema screen should omit a reference, and, if possible, a film describing the work and needs of the Metropolitan. The work already done, and the goodwill which it shows, encourage us to repeat these far from novel suggestions. The truth is methods do not change. The variable factor is the degree of thoroughness with which they are carried out.

### HOSPITAL ACTIVITY AT BANGOR

At their annual meeting on Monday the Governors of the Carnarvonshire and Anglesey Infirmary at Bangor decided to make a public appeal for £50,000 for the purpose of building and endowing a new hospital. The British Red Cross have made a grant of £17,500 to the building fund, and it is announced that Mr. J. R. Davis, of Ceres, has given a donation of £1,000 to the endowment fund. A deficit of £1,026 was reported on the year's working. It was decided to increase the in-patients' fees from 7s. 6d. to 12s. 6d. a week.

### A GOLD WATCH FOR THE RED CROSS SECRETARY.

Sir Basil Mayhew, who has been the Secretary of the Joint Finance Committee of the British Red Cross Society and the Order of St. John since it was established, has been presented with a gold watch and chain by the members of the Committee. The presentation was made at a dinner at the Savoy Hotel, when Sir Robert Hudson presided over a gathering of past and present members of the Committee, of which he has been Chairman since the outbreak of the war. There were present the Earl of Plymouth, the Hon. Sir Arthur Stanley, the Right Hon. Evelyn Cecil, M.P., the Hon. Sir Charles Russell, the Hon. Sir William Goschen, Sir William Garstin, Sir Herbert Jekyll, Sir Aurelian Redsdale, Sir Herbert Perrott, and Mr. J. Danvers Power. The only other guest at the dinner was Viscount Northcliffe, in special recognition of the great help rendered by *The Times* in raising the Joint Committee's funds.

### A HOTEL-KEEPER'S APPEAL.

Mr. Harry Preston, of the Royal York Hotel, Brighton, has a bright idea which he is trying to put to advantage on behalf of the Royal Sussex Hospital, Brighton. He says that it has occurred to him that there must be many thousands of people who have regained health by visiting the favourite south-coast resort, and, "having great belief that there is at least a grain of gratitude within the hearts of every person," he suggests that every one of those people who have been patients of Dr. Brighton should send him just £1 Treasury note, or send it direct to the hospital, for the Harry Preston £1 Note Hospital Fund in aid of the Royal Sussex Hospital. All subscriptions will be acknowledged by the Chairman of the hospital, which had a large deficit last year. We hope that Mr. Preston's appeal will go far to put matters right. This is the way to go to work—to interest people who have learned by experience what health means to happiness and to make them think of those who are at the present time in exactly the same position that they were in at one time themselves.

### THIS WEEK'S DRUG MARKET.

The market is still depressed and there are no signs of immediate improvement in sight. The downward movement of prices has not been checked and buyers are still limiting their purchases to their immediate requirements, which would seem to be a wise policy. The mania for pushing up prices which seems to have seized everybody who has anything to sell had attacked the drug market in a particularly severe way and this sudden resentment on the part of buyers is the proper treatment for the prevalent complaint. One finds not uncommonly that the produce markets set the fashion to other markets, and buyers of hospital stores of all kinds would be well advised to bear this in mind. Statesmen and others talk glibly about the probabilities of the price of a suit of clothes running up to £20, and of printing paper reaching a figure far beyond the present infamous price; they may be true prophets or they may be false ones. Not many weeks ago there were plenty of people "in the know" who thought menthol, camphor, peppermint oil, shellac, and many other things were going to keep on moving upwards almost indefinitely. They were wrong.

